# Phase II INTERACT-ION study: ezabenlimab (BI 754091) and mDCF (docetaxel, cisplatin, and 5-fluorouracil) followed by chemoradiotherapy in patients with Stage III squamous cell anal carcinoma

**DOI:** 10.3389/fonc.2022.918499

**Published:** 2022-08-24

**Authors:** Stefano Kim, Jihane Boustani, Dewi Vernerey, Véronique Vendrely, Ludovic Evesque, Eric Francois, Laurent Quero, Francois Ghiringhelli, Christelle de la Fouchardière, Laëtitia Dahan, Oliver Bouché, Benoist Chibaudel, Farid El Hajbi, Chloé Vernet, Magali Rebucci-Peixoto, Alexandra Feuersinger, Christophe Maritaz, Christophe Borg

**Affiliations:** ^1^Department of Medical Oncology, University Hospital of Besançon, Besançon, France; ^2^Clinical Investigational Center, INSERM CIC-1431, Centre Hospitalier Universitaire de Besançon, Besançon, France; ^3^INSERM, Unit 1098, University of Bourgogne Franche-Comté, Besançon, France; ^4^Department of Radiotherapy, University Hospital of Besançon, Besançon, France; ^5^Methodology and Quality of Life in Oncology Unit, University Hospital of Besançon, Besançon, France; ^6^Department of Radiation Oncology, Bordeaux University Hospital, Pessac, France; ^7^Departement of Oncology, Centre Antoine Lacassagne, Nice, France; ^8^INSERM, Unit 1160, University of Paris, Paris, France; ^9^Department of Radiation Oncology, Saint-Louis Hospital, APHP, Paris, France; ^10^Department of Medical Oncology, Centre Georges-François Leclerc, Dijon, France; ^11^Department of Medical Oncology, Centre Léon Bérard, Lyon, France; ^12^Department of Digestive Oncology, La Timone, Aix Marseille Université, Marseille, France; ^13^Department of Digestive Oncology, Hôpital Robert Debré, Reims, France; ^14^Department of Medical Oncology, Hôpital Franco-Britannique, Fondation Cognacq-Jay, Levallois-Perret, France; ^15^Department of Oncology, Centre Oscar Lambret, Lille, France; ^16^Department of Digestive Oncology, Hôpital Privé Jean Mermoz, Lyon, France; ^17^Global Medical Affairs, Oncology, Boehringer Ingelheim International GmbH, Ingelheim am Rhein, Germany; ^18^Medical Affairs Department, Oncology, Boehringer Ingelheim France, Paris, France

**Keywords:** ezabenlimab, squamous, anal carcinoma, modified Docetaxel, Cisplatin, 5-Fluorouracil (DCF), Stage III

## Abstract

**Background:**

Chemoradiotherapy alone is the standard treatment for locally advanced squamous cell anal carcinoma (SCAC). However, up to 50% of patients will experience recurrence; thus, there is a need for new treatments to improve outcomes. Modified docetaxel, cisplatin and 5-fluorouracil (mDCF) is a treatment option for first-line metastatic SCAC, having shown efficacy in the Epitopes-HPV01 and -02 trials (NCT01845779 and NCT02402842). mDCF treatment also plays a role in the modulation of anti-tumor immunity, suggesting it may be a good combination partner for immunotherapy in patients with SCAC. Anti-programmed death protein-1 (PD-1) immunotherapy has been shown to be effective in metastatic SCAC. We therefore designed the INTERACT-ION study to assess the combination of mDCF with ezabenlimab (BI 754091), an anti-PD-1 antibody, followed by chemoradiotherapy, in patients with Stage III SCAC.

**Methods:**

INTERACT-ION is a pivotal, open-label, single-arm phase II study in patients with treatment-naïve Stage III SCAC. Patients will receive induction treatment with mDCF (docetaxel 40 mg/m^2^ and cisplatin 40 mg/m^2^ on Day 1, 5-fluorouracil 1200 mg/m^2^/day for 2 days) every 2 weeks for 4 cycles and ezabenlimab (240 mg given intravenously) every 3 weeks for 3 cycles. In the absence of disease progression at 2 months, two additional cycles of mDCF and one additional cycle of ezabenlimab will be administered. Patients with radiological objective response, pathological complete/near-complete response and biological complete response will then receive an involved-node radiotherapy with intensity-modulated radiation therapy and concurrent chemotherapy, followed by ezabenlimab alone for seven cycles. All other patients will receive standard chemoradiotherapy. The primary endpoint is the clinical complete response rate 10 months after the first cycle of mDCF plus ezabenlimab. Major secondary endpoints are major pathological response and biological complete response after induction treatment. An extensive ancillary biomarker study in tumor tissue and peripheral blood will also be conducted.

**Discussion:**

The addition of immunotherapy to chemotherapy is an area of active interest in metastatic anal cancer. This pivotal study will evaluate this combination in the locally advanced setting. Ancillary biomarker studies will contribute to the understanding of predictors of response or resistance to treatment.

**Clinical Trial Registration:**

https://clinicaltrials.gov/ct2/show/NCT04719988, identifier NCT04719988.

## Background

Anal cancer is a rare disease, accounting for less than 1% of all new cancer cases; however, its incidence is increasing in Europe, Australia, and the United States (1). In particular, there have been notable increases in patients being diagnosed with Stage III and IV disease (2, 3). The most common histological subtype (~85% of cases) is squamous cell anal carcinoma (SCAC) (4). There is a strong association between SCAC and infection with a high-risk form of human papillomavirus (HPV), such as HPV-16 (5). For patients with locally advanced SCAC, mitomycin C and 5-fluorouracil (5-FU)-based chemoradiotherapy is standard treatment (6–8). However, around 25–40% of patients will experience disease recurrence (9), and recurrence rates may be as high as 50% at 3 years in patients with T3 or greater disease and/or nodal involvement (10, 11). As such, there is a need for treatments that can improve outcomes in these patients.

Modified docetaxel, cisplatin and 5-FU (mDCF) is an option for first-line metastatic SCAC based on data from the Epitopes-HPV01 and 02 trials (12, 13). mDCF was associated with median progression-free survival (PFS) of 11.0 months (95% confidence interval [CI] 6.8–16.4) in the Epitopes-HPV02 study. Of 30 patients who received mDCF, 14 (47%) had a complete response and 25 (83%) had an objective response (12). Pooled analyses of Epitopes-HPV01 and 02 confirmed the efficacy of the mDCF regimen (median PFS of 14.5 months [95% CI 10.1–18.9]) (13). Interestingly, 29 chemotherapy-naïve patients in these pooled analyses had synchronous metastases (detected at or shortly after diagnosis of the primary tumor). Among these patients, objective response rate (ORR) was 90%, including 55% with a radiological complete response, and median PFS was 16.4 months. This suggests a potential role for mDCF as an induction treatment in locally advanced SCAC.

Docetaxel has been shown to be involved in modulating anti-tumor immune responses (14) and results from ancillary studies of Epitopes-HPV01 and HPV-02 provide further support for this mechanism. Monocytic myeloid-derived suppressive cells (M-MDSCs) were increased in peripheral blood from patients with SCAC versus healthy donors (14). DCF treatment led to a reduction in M-MDSC levels; furthermore, high M-MDSC levels (pre- or post-treatment with DCF) were associated with shorter overall survival (OS) in patients with SCAC. Telomerase reverse transcriptase (TERT) is another marker of interest in SCAC, as the HPV oncoprotein E6 transactivates TERT (15). In Epitopes-HPV01 and HPV02, DCF treatment was associated with an increase in anti-hTERT immunity, particularly in patients who had low levels of M-MDSCs (14). Moreover, anti-hTERT T-Helper 1 CD4 responders had improved PFS versus non-responders (12). We therefore considered that mDCF could be a good combination partner for immunotherapy in patients with SCAC. Anti-programmed death protein-1 (PD-1) therapy has been shown to be effective in patients with previously treated metastatic SCAC (16, 17). Furthermore, anti-PD-1 therapy has shown efficacy as a neoadjuvant therapy in non-small cell lung cancer (18), bladder cancer (19), and melanoma (20), with complete or major pathological responses observed in 30–45% of patients.

Ezabenlimab (BI 754091), an anti-PD-1 antibody, has been investigated as monotherapy and is currently being investigated in combination with other anti-cancer therapies. The recommended Phase II dose has been determined as 240 mg given intravenously every 3 weeks and data from early phase trials suggest that the efficacy and safety profile of ezabenlimab is consistent with that of other anti-PD-1 or anti-programmed death-ligand 1 (PD-L1) antibodies (21).

We designed the following Phase II INTERACT-ION study to assess the combination of mDCF with ezabenlimab (BI 754091) followed by chemoradiotherapy (CRT).

The feasibility of combining a mDCF and immunotherapy has recently been demonstrated in advanced disease in the SCARCE-PRODIGE 60 study (22). In this study, the median number of cycles was 8 of 8 scheduled cycles (interquartile range [IQR] 8.0–8.0) for mDCF and 16 cycles (IQR 9.5–16.0) for the anti-PD-L1 molecule. Dose compliance for the cycles was almost 100% for mDCF, and 100% for the anti-PD-L1 molecule. Grade 3–4 adverse events were observed in 59.0% of patients, and were mostly hematological (anemia 16.4%, neutropenia 14.8%). Only one patient (1.6%) presented febrile neutropenia. The feasibility of CRT after DCF was also demonstrated in a retrospective analysis of three prospective trials in advanced SCAC disease (Grave, A. et al. Frontiers in Oncology. In Press). In this study, 16 patients received CRT after 6 cycles of classic DCF or 8 cycles of mDCF. All patients received the complete radiation dose.

We hypothesize that the combination of mDCF, ezabenlimab and radiotherapy may act synergistically to improve treatment outcomes. The primary objective is to assess the clinical complete response (cCR) rate 10 months after the first cycle of mDCF and ezabenlimab. An extensive ancillary biomarker study in tumor tissue and peripheral blood will also be conducted to further understand potential synergistic effects between mDCF, immunotherapy and radiotherapy, and to evaluate predictors of response or resistance to treatment.

## Methods and analysis

### Study design

INTERACT-ION is a Phase II, open-label, pivotal, single-arm study of ezabenlimab and the mDCF regimen followed by chemoradiotherapy in patients with Stage III SCAC (NCT04719988). The study is sponsored by the Centre Hospitalier Universitaire de Besançon and will be conducted at twelve centers in France. Details of study sites can be obtained *via* www.clinicaltrials.gov. The study has received a favorable opinion from the Ethics Committee Sud-Méditerranée I and authorization from the French National Agency for the Safety of Medicines and Health Products and will be conducted in accordance with Good Clinical Practice guidelines.

### Eligibility criteria

Adult patients with treatment-naïve, locally advanced, histologically proven Stage III SCAC and an Eastern Cooperative Oncology Group performance status of 0 or 1 will be enrolled. Full details of inclusion and exclusion criteria are shown in **Table 1**.

**Table 1:** Inclusion and Exclusion Criteria.

Key Inclusion Criteria
Histologically confirmed squamous cell anal carcinoma
Locally advanced disease (defined as Stage III [TxN1 or T4N0])
Age ≥18 years
ECOG PS of 0 or 1
CT scan, MRI of pelvis and PET scan performed within 30 days prior to inclusion
Adequate organ function (hematological, renal, hepatic, coagulation) within 7 days of treatment initiation
Affiliated to or beneficiary of French social security health insurance system
**Key Exclusion Criteria**
Prior chemotherapy, pelvic radiotherapy, or anti-tumor immunotherapy
Metastatic disease
Diagnosis of additional malignancy within 3 years prior to the inclusion date, with the exception of curatively treated basal cell carcinoma of the skin and/or curatively resected *in situ* cervical or breast cancer
Any medical or psychiatric condition or disease which would make the patient inappropriate for entry into the study
Current participation in a study of an investigational agent
Pregnancy, breast-feeding, or absence/refusal of adequate contraception for fertile patients during the period of treatment and for 6 months from the last treatment administration
Under guardianship, curatorship, or under protection of justice
***Non-eligibility for chemotherapy***
Inadequate organ function
Diabetes with vascular or neurovascular complications
Pre-existent peripheral neuropathy or impaired audition
HIV-positive patients with CD4+ cell count **<**400/mm^3^ (HIV test is mandatory before inclusion)
Active hepatitis B or C virus infection
Active tuberculosis
Concomitant treatment with CYP3A4 inhibitor
Known hypersensitivity or contraindication to any of the study chemotherapy drugs and dihydro pyrimidine dehydrogenase deficit
Uncontrolled infection or another life-threatening condition
Administration of a live vaccine with 28 days of planned start of study therapy
Administration of prophylactic phenytoin
Inadequate laboratory values: MDRD CrCL **<**60 ml/min, neutrophil count **<**1500/mm^3^, platelets **<**100,000/mm^2^, bilirubin 2.5 x ULN, AST/ALT 2.5 x ULN
Major surgery (requiring general anesthesia) within 28 days of enrollment
***Non-eligibility for immunotherapy***
Any immunosuppressive therapy within 14 days before the planned start of therapy
Active autoimmune disease that has required a systemic treatment in the past 2 years
Prior allogeneic bone marrow transplantation or prior solid organ transplantation
Known active central nervous system metastases and/or carcinomatous meningitis
Previously received an anti-PD-1, anti-PD-L1 or anti-CTLA4 agent
Known hypersensitivity or allergy to Chinese hamster ovary cell products or any component of ezabenlimab formulation
History of colorectal inflammatory disease
History of idiopathic or secondary pulmonary fibrosis or evidence of active pneumonitis requiring a systemic treatment within 28 days before the planned start of therapy
History of severe hypersensitivity reactions to other monoclonal antibodies
***Non-eligibility for radiotherapy***
History of chronic colorectal inflammatory disease
History of connective tissue disease
History of autoimmune disease

ALT, alanine aminotransferase; AST, aspartate aminotransferase; CrCL, creatinine clearance; CT, computed tomography; CYP3A4, cytochrome P450 3A4; ECOG PS, Eastern Cooperative Oncology Group performance status; HIV, human immunodeficiency virus; MDRD, modification of diet in renal disease; MRI, magnetic resonance imaging; PD-1, programmed death protein-1; PD-L1, programmed death-ligand 1; PET, positron emission tomography; ULN, upper limit of normal.

### Study treatments

**Figure 1:**
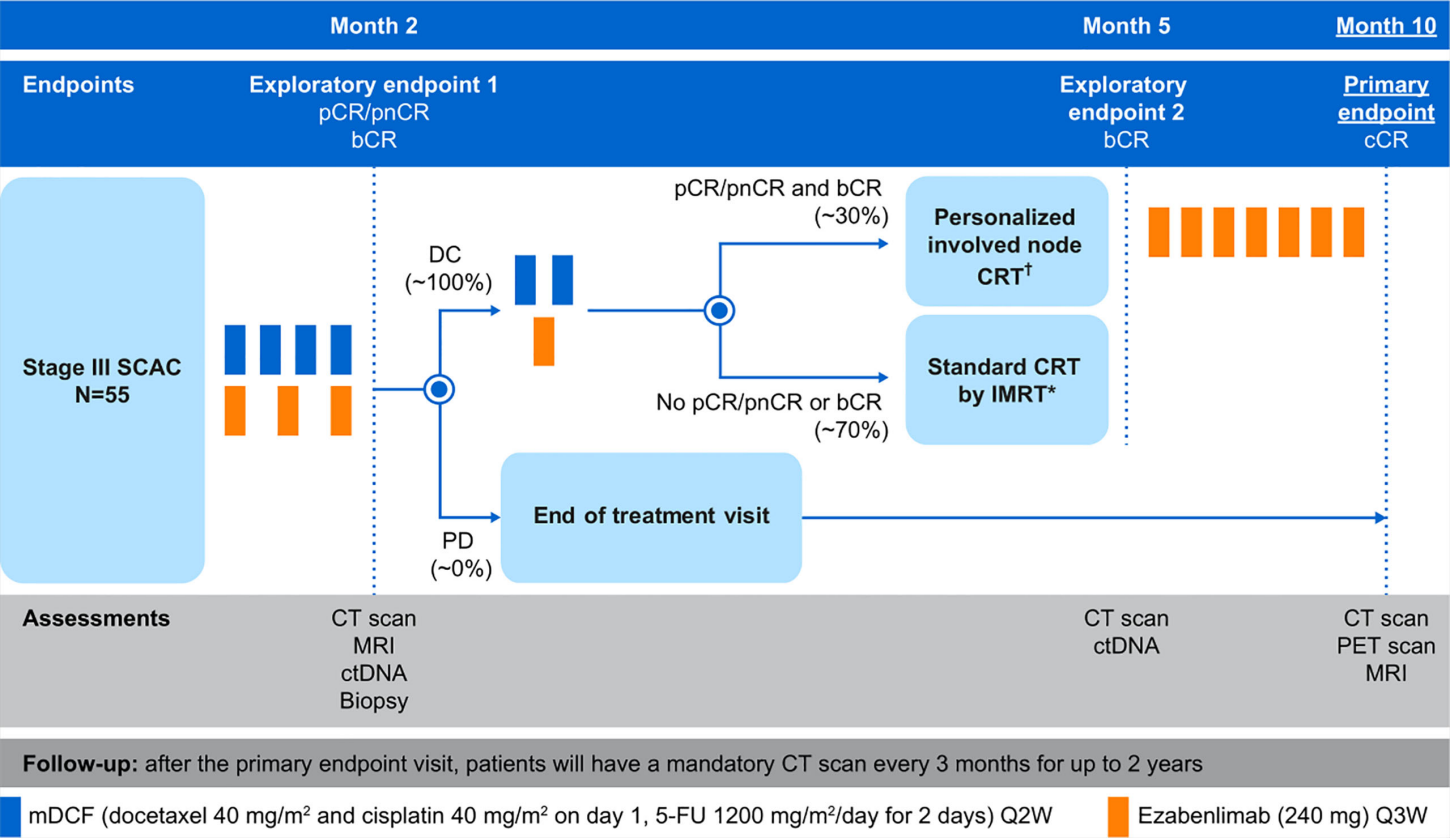
Study Design of INTERACT-ION. *Concomitantly given with capecitabine (825 mg/m^2^ orally twice daily) only on days of radiotherapy, and mitomycin C (10 mg/m^2^ Day 1 ± Day 29). ^†^59.4 Gy for primary tumor and involved residual nodes + 45 Gy for elective nodal irradiation for initially involved lymph node(s) with complete response. 5-FU, 5-fluorouracil; bCR, biological complete response; cCR, complete clinical response; CT, computed tomography; ctDNA, circulating tumor DNA; DC, disease control; IMRT, intensity-modulated radiation therapy; MRI, magnetic resonance imaging; pCR, pathological complete response; PD, progressive disease; PET, positron emission tomography; pnCR, pathological near-complete response; Q2W, every 2 weeks.

Patients will receive induction treatment with mDCF every 2 weeks for 4 cycles (docetaxel 40 mg/m^2^, day 1; cisplatin 40 mg/m^2^, day 1; 5-FU, 1200 mg/m^2^/day, days 1 and 2) and ezabenlimab (240 mg given intravenously every 3 weeks for 3 cycles; **Figure 1**). Systematic primary prophylaxis with granulocyte-colony stimulating factor from the first cycle is permitted at the investigator’s discretion. No premedication is indicated for administration of the first cycle of ezabenlimab; however, patients who experience an infusion-related reaction during the first cycle may receive premedication (antihistamines or antipyretics/analgesics, such as acetaminophen) for subsequent infusions. In the absence of tumor progression at 2 months, two additional cycles of mDCF and one additional cycle of ezabenlimab will be administered. If the patient has experienced disease progression (>20% per Response Evaluation Criteria in Solid Tumors [RECIST] v1.1), patients will have an end of treatment visit and will receive standard treatment per their institution. These patients will also complete the primary endpoint visit at Month 10.

**Figure 2:**
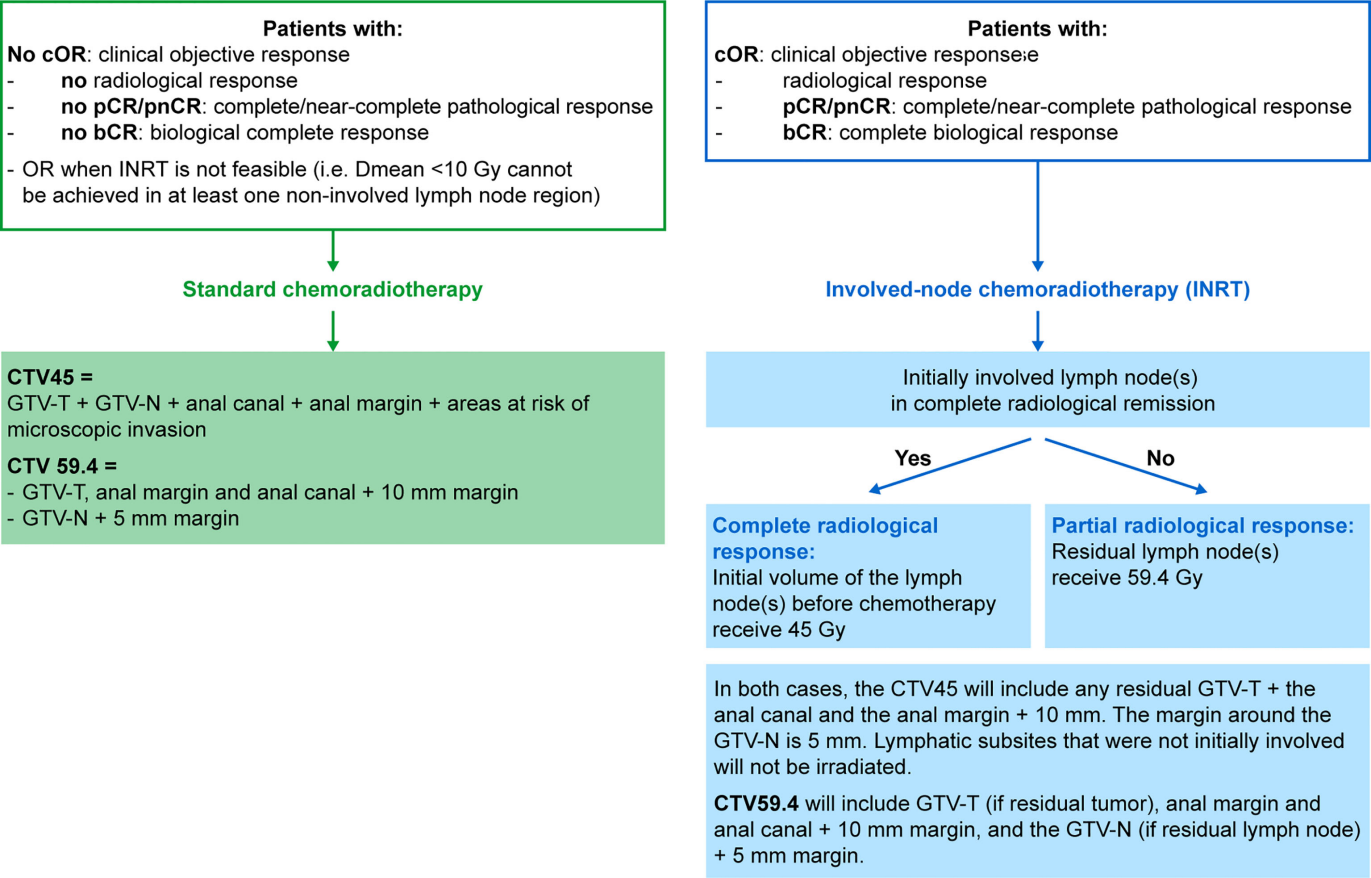
Flow Chart of Chemoradiotherapy. CTV, clinical target volume; Dmean, mean dose; GTV, gross tumor volume; Gy, Gray.

After the additional cycles of mDCF and ezabenlimab, subsequent treatment will depend on assessment of pathology and biological results. Patients with radiological objective response (≥30% by RECIST 1.1), pathological complete or near complete response (viable tumor cells/tumor bed at biopsy ≤10%) and biological complete response (no residual HPV circulating tumor DNA [ctDNA]) will receive chemoradiotherapy by intensity-modulated radiation therapy (IMRT) with an involved-node radiotherapy (INRT; **Figure 2**). For INRT, no elective nodal irradiation will be performed; however, all different lymphatic subsites should be delineated separately. INRT will be performed if at least one non-involved lymphatic subsite among inguinal nodes, external iliac node, obturator and internal iliac nodes and the presacral space receive a mean dose <10 Gy. Otherwise, patients will receive standard chemoradiotherapy. If the initially involved lymph node(s) are no longer visible or of normal size (i.e. complete radiological response), the clinical target volume (CTV) 45 is defined as the initial volume of the lymph node(s) before chemotherapy and the prescribed dose is 45 Gy. Normal structures that have been displaced by the enlarged lymph node(s) are not included in the irradiated volume. Wherever possible, blood vessels are not included in the CTV if the involved lymph nodes are clearly located at a distance from them. If the initially involved lymph node(s) are in partial remission, the residual lymph node(s) will receive 59.4 Gy. In both cases of complete and partial radiological response, the CTV45 will include any residual gross tumor volume-tumor (GTV-T) plus the anal canal and the anal margin plus 10 mm. The margin around the GTV-node (GTV-N) is 5 mm. Lymphatic subsites that were not initially involved will not be irradiated. CTV59.4 will include GTV-T (if residual tumor), anal margin and anal canal plus a 10 mm margin, and the GTV-N (if residual lymph node) plus a 5 mm margin. Radiotherapy will be given concomitantly with capecitabine (825 mg/m^2^ orally, twice daily on days of radiotherapy) and mitomycin C (10 mg/m^2^ by intravenous infusion on Day 1 ± Day 29 [at the discretion of the investigator]). Patients will then receive ezabenlimab alone (240 mg every 3 weeks) for 7 cycles after completion of radiotherapy (3–6 weeks post radiotherapy).

All other patients will receive standard chemoradiotherapy (**Figure 2**). Chemoradiotherapy using IMRT will commence 3–4 weeks after the last cycle of mDCF and ezabenlimab and will be given concomitantly with capecitabine (825 mg/m^2^ orally, twice daily only on days of radiotherapy) and mitomycin C (10 mg/m^2^ by intravenous infusion on Day 1 ± Day 29). Following completion of chemoradiotherapy, patients will be monitored (surgery is possible if there is residual disease after the primary endpoint assessment).

Dose modification and interruption for mDCF, capecitabine and mitomycin will be managed per their respective Summary of Product Characteristics. Dose modification of ezabenlimab is not permitted; however, ezabenlimab treatment can by delayed for a maximum of 2 cycles for the management of adverse events (AEs).

To date, there is no international consensus on the optimum dose of radiotherapy to deliver. Based on the ESMO guidelines (6), the total doses vary between countries from 50.4 Gy as used in the ACT II trial (8), 55–59 Gy for T3–4 or node-positive disease as used in the RTOG 98-11 trial (11), and up to 64 Gy used in marge series from the Nordic counties (23). Furthermore, tumor control probability models suggest that lower doses may be sufficient for small tumors, while higher doses (50–55 Gy or higher) may be required for more advanced tumors such as T3–4 or N1. In the recently updated guidelines of the French Society for Radiation Oncology (24), the standard dose is 45 Gy in 25 fractions over five weeks, delivered to the first planning target volume corresponding to the tumor, the ilio-obturator nodes and pelvic and inguinal nodal areas. A localized irradiation boost to the second planning target volume (primary tumor and involved nodes) delivers 15–20 Gy without interrupting treatment. Thus, the recommended total dose to initially invaded sites is 60 Gy over six weeks or the equivalent in 1.8–2 Gy per fraction.

In the current study, we focus on stage III anal cancer and chose to deliver 45 Gy to nodal areas and 59.4 Gy to the primary tumor and involved nodes. In the control arm, a standard chemoradiation is delivered, including elective irradiation of the following areas: external and internal iliac areas, ilio-obturator areas, perirectal area, inguinal areas, presacral area, and ischiorectal fossa.

Radiation-induced lymphopenia (RIL) is a well-recognized phenomenon that can develop in up to 70% of patients undergoing radiotherapy (25), especially when pelvic bone structures are irradiated. RIL is characterized by acute preferential depletion of CD4+T-cells and B-cells (26). Several studies have established RIL as a negative prognostic factor (27). Furthermore, lymphopenia can reduce the efficacy of immune checkpoint inhibitors (28). In this context, the standard irradiation of the whole pelvis including all lymph node fields could eliminate the beneficial effect of maintenance immunotherapy in responding patients. Thus, in the experimental arm, the dose delivered to the primary tumor and the involved residual nodes will remain the same (59.4 Gy). However, elective nodal irradiation will not be performed. If initially involved lymph node(s) are no longer visible or of normal size, a dose of 45 Gy will be delivered to the initial volume of the lymph node(s) before chemotherapy. This will be performed only in patients with objective clinical and radiological response, and complete/near-complete pathological response, and complete biological response.

### Study endpoints

The primary endpoint is the cCR rate 10 months after treatment initiation (**Figure 1**). Based on data from the ACTII study, the optimal timepoint for assessment of tumor response after standard chemoradiotherapy was 26 weeks (29); an additional 14 weeks are necessary in our protocol due to the neoadjuvant treatment stage. The main secondary endpoints include major pathological response (pathological complete response) or pathological near-complete response after induction treatment (after 4 cycles of mDCF and 3 cycles of ezabenlimab) and biological complete response (defined as non-detectable HPV ctDNA) after i) induction treatment and ii) 4 weeks after the completion of radiotherapy. Other secondary endpoints are: ORR; OS; median PFS and PFS rates at 2 and 3 years; median recurrence free-survival (RFS) and RFS rates at 2 and 3 years; impact of treatment on patient’s health-related quality of life (HRQoL); safety of combination treatment; positron emission tomography (PET)-computed tomography (CT) complete response (defined as the absence of pathological hypercaptation); PET-CT complete response will be correlated to cCR, RFS and PFS.

Endpoints for the ancillary biomarker study are: correlation of peripheral CD4 anti-HPV and anti-telomerase immunity as well as regulatory and MDSCs with PFS; HPV and telomerase-specific T cell responses before and after treatment; characterization of tumor genotyping for HPV and p53; tumor-infiltrating lymphocytes and PD-L1 expression in tumors; correlation of PD-L1 and PD-L2 immunohistochemistry with PFS; prognostic value of circulating HPV ctDNA using polymerase chain reaction on cell-free tumor DNA; predictive value of soluble markers from plasma and enzyme-linked immunosorbent assay (ELISA).

### Study schedule and assessments

**Figure 3:**
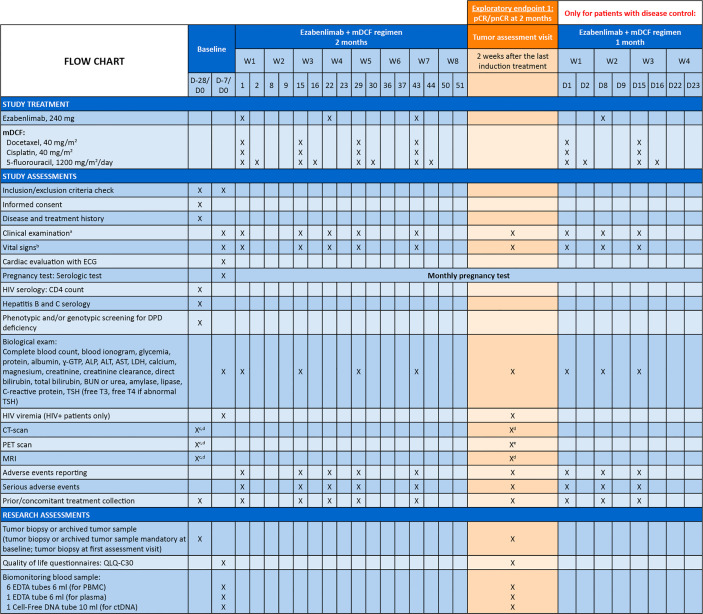
Study Flow Chart and Visit Schedule. ^a^Clinical examination: height (only at baseline), weight, Eastern Cooperative Oncology Group performance status; ^b^Vital signs: pulse, blood pressure and body temperature; ^c^Only if no radiologic assessment performed within 28 days prior to this visit; ^d^Radiologic assessment mandatory; ^e^Radiologic assessment highly recommended; ^f^Radiologic assessment if indicated ALP, alkaline phosphatase; ALT, alanine aminotransferase; AST, aspartate aminotransferase; bCR, biological complete response; BUN, blood urea nitrogen; cCR, clinical complete response; cOR, clinical objective response; CT, computed tomography; ctDNA, circulating tumor DNA; CTV, clinical target volume; D, day; DPD, dihydropyrimidine dehydrogenase; ECG, electrocardiogram; EDTA, ethylenediaminetetraacetic acid; F, Friday; γ-GTP, gamma-glutamyl transpeptidase; GTV, gross tumor volume; Gy, Gray; HIV, human immunodeficiency virus; IMRT, intensity-modulated radiation therapy; INRT, involved-node radiotherapy; LDH, lactate dehydrogenase; mDCF, modified docetaxel, cisplatin and 5-fluorouracil; MRI, magnetic resonance imaging; PBMC, peripheral blood mononuclear cells; pCR, pathologic complete response; PET, positron emission tomography; pnCR, pathologic near complete response; TSH, thyroid stimulating hormone; W, week.

The study flow and visit schedule are shown in **Figure 3**. Tumor assessments will be performed at specific timepoints during the study. At baseline, patients will have a mandatory CT scan, PET scan and magnetic resonance imaging (MRI). At 2 months (after induction treatment), patients will have a mandatory CT scan and MRI, and a PET scan is highly recommended. Patients will have further CT scans at 5 months (4 weeks after the completion of radiotherapy) and at the end of treatment visit. At the primary endpoint visit (10 months), patients will have a mandatory CT scan and MRI, and a PET scan is highly recommended. Thereafter, patients will have a mandatory CT scan at each follow-up visit (every 3 months for up to 2 years). Patients may also have a PET scan and MRI if indicated.

Response will be evaluated by the investigator per RECIST version 1.1. For patients who discontinue treatment for reasons other than documented disease progression, loss to follow-up or consent withdrawal, tumor assessment must continue every 8 weeks during the first year and every 12 weeks thereafter until documented disease progression. Radiological assessments will be anonymized by the investigational centers and all imaging data will be collected at the end of the study for centralized review.

Safety will be assessed throughout the study by evaluation of AEs, clinical safety laboratory tests (blood analysis, cardiac evaluation), vital signs, and physical examinations. Investigators will evaluate the intensity of AEs (per National Cancer Institute Common Terminology Criteria for Adverse Events version 5) and any causal relationship to study treatment.

HRQoL will be assessed by completion of the European Organisation for Research and Treatment of Cancer core quality of life questionnaire (QLQ-C30) at baseline, evaluation visits, end of treatment visit, endpoint visit, and at follow-up visits (every 3 months after the endpoint visit).

A tumor biopsy or archived tumor sample will be mandatory at baseline (for assessment of tumor-infiltrating lymphocyte levels, checkpoint expression [including PD-L1, LAG-3, TIM-3, TIGIT] and to perform a nanostring assay for RNA expression, including interferon signature). A mandatory fresh tumor biopsy will also be taken at the first tumor assessment visit to measure lymphocyte infiltration levels, immune checkpoint expression, pathological response, and perform a nanostring assay of RNA expression. Biomonitoring blood samples will be taken at baseline, first tumor assessment, and after chemoradiotherapy. Peripheral blood mononuclear cells will be used to phenotype T cell lymphocytes, MDSCs, monocytes, and antigen-specific T cells. Plasma will be analyzed to monitor immune and angiogenic-related biomarkers by ELISA, and PD-L1 and immune checkpoint inhibitor ligand expression. ctDNA will be used to monitor HPV-derived oncoprotein DNA (E6 and E7).

### Monitoring and safety

As the safety profile of ezabenlimab and mDCF has not been evaluated in Stage III SCAC, semi-continuous monitoring for toxicity using a Pocock-type boundary will also be performed (targeted dose-limiting toxicity [DLT] rate: 0.20). Any non-hematologic grade 4 toxicity lasting >1 week, any immune-related grade 3/4 toxicity lasting >1 week, and hyperprogression will be considered as DLTs. An independent Data Safety Monitoring Board (DSMB) will meet to monitor toxicities and review serious AEs every 6 months or if the DLT number exceeds the boundary limit.

### Statistics

#### Determination of sample size

cCR rate at 26 weeks after starting radiotherapy was achieved in 80% of all patients in the ACTII study. Among patients with locally advanced disease, cCR at 26 weeks was approximately 70% in patients with T3/T4 disease and approximately 65% in patients with node-positive disease (29). As such, the following hypotheses will be considered: H0 (null) where a cCR rate of 65% at 10 months is uninteresting and H1 (alternative) where a cCR rate of 81% at 10 months is expected.

Per A’Hern (with a one-sided 5% type I error and power of 80%), 52 evaluable patients for cCR at 10 months will be required to test the hypothesis. There is an expected 5% rate of patients not being evaluable or lost to follow-up at 10 months, necessitating a planned sample size of 55 patients.

### Planned analyses

The primary analysis will be performed on the modified intention-to-treat population (mITT) which will include all evaluable patients who receive at least one cycle of treatment. Safety will be assessed in patients who received any study medication, and HRQoL will be assessed in patients within the mITT population who complete at least one questionnaire at baseline.

### Trial status

The INTERACT-ION trial is open for recruitment. The first patient was enrolled in December 2021, after all legal approvals required in France were received.

## Discussion

Current standard treatment for locally advanced SCAC is mitomycin C and 5-FU-based chemoradiotherapy (6); however, recurrence rates may be as high as 50% at 3 years (10, 11). mDCF has been shown to be effective for the first-line treatment of metastatic SCAC (12, 13), and docetaxel also appears to enhance immune responses (14), suggesting that mDCF is an attractive candidate to combine with immunotherapy.

This paper describes the protocol of a Phase II, open-label, pivotal, single-arm study of ezabenlimab and the mDCF regimen followed by chemoradiotherapy in patients with Stage III SCAC. An extensive ancillary study will also assess predictors of response or resistance to treatment.

The combination of chemotherapy and immunotherapy is an area of active research in metastatic anal cancer (30). Of note, two ongoing Phase III studies are assessing chemotherapy in combination with PD-1 inhibition. Firstly, the combination of carboplatin and paclitaxel with nivolumab will be assessed in approximately 205 treatment-naïve patients with metastatic anal cancer (NCT04444921). Secondly, the combination of carboplatin and paclitaxel with retifanlimab will be evaluated in around 300 patients with inoperable, locally recurrent or metastatic SCAC (NCT04472429). Both of these trials are currently recruiting patients. Additionally, the Phase II SCARCE trial (NCT03519295) is assessing mDCF in combination with the anti-PD-L1 antibody, atezolizumab (9). The INTERACT-ION study, presented here, as well as the trials being conducted in the metastatic setting, should contribute further information on the treatment of locally advanced and metastatic SCAC, and the optimal chemotherapy regimens and immunotherapy combination partners.

## Ethics statement

The study received a favorable opinion from the Ethics Committee (CPP) Sud-Méditerranée I and authorization from the French National Agency for the Safety of Medicines and Health Products.

## Author contributions

SK, JB, DV, MR-P, CM and CB were involved in the conception and design of the study. SK, JB, VV, LE, EF, LQ, FG, CF, LD, OB, BC, FE, CV, and CB are involved in patient recruitment and provision of materials. SK, JB, DV, MR-P, AF, and CB are involved in data analysis and interpretation. All authors were involved in the drafting of the manuscript and/or critically revised the manuscript for important intellectual content. All authors gave final approval of the manuscript and agree to be accountable for all aspects of the work, which includes ensuring that questions related to the accuracy or integrity of any part of the work are appropriately investigated and resolved.

## Acknowledgments

The authors received no direct compensation related to the development of the manuscript. The authors would like to thank Dr Edward Espinal-Dominguez for his contribution to an early draft of the manuscript. Medical writing support for the development of this manuscript, under the direction of the authors, was provided by Caroline Allinson and Hannah Simmons MSc of Ashfield MedComms, an Ashfield Health Company, and was funded by Boehringer Ingelheim.

## Funding

The study is sponsored by Centre Hospitalier Universitaire de Besançon. The study is funded by the sponsor and Boehringer Ingelheim. BI were involved with the study design, collection, analysis, interpretation of data, the writing of this article or the decision to submit it for publication.

## Conflict of interest

SK reports a consulting or advisory role for Ipsen, Incyte, Boehringer Ingelheim, Sanofi and BeiGene and has received research funding from Pfizer, Roche, Novartis, Bristol-Myers Squibb, Boehringer Ingelheim and Sanofi. LE reports honoraria from Servier, Merck, Pierre Fabre and Amgen and travel and accommodation expenses from Merck and Servier. EF reports a consulting or advisory role for Pierre Fabre; has received travel and accommodation expenses from Pierre Fabre and honoraria from Amgen, Novartis, Pierre Fabre, and Merck Sharp & Dohme. LQ reports research funding from AstraZeneca, and travel and accommodation expenses from Astellas and Ipsen. CF reports a consulting or advisory role for Amgen, Bristol-Myers Squibb, Eisai, Pierre Fabre Oncologie, Roche, Servier and Ipsen; and travel and accommodation expenses from Eisai and Amgen. LD reports honoraria from Amgen, Bristol-Myers Squibb, Servier, Oseus and Mylan. OB reports honoraria from Roche, Amgen, Merck Serono, Servier, Pierre Fabre, Bayer, Sanofi and Grünenthal. BC reports a consulting or advisory role for BeiGene, Roche, Sanofi and Servier; travel and accommodation expenses from Sanofi, Merck, Merck Sharp & Dohme and Roche; and honoraria from Pfizer, Roche, Sanofi, Servier and Bayer. AF and CM are employees of Boehringer Ingelheim.

The remaining authors declare that the research was conducted in the absence of any commercial or financial relationships that could be construed as a potential conflict of interest.

The authors declare that this study received funding from Boehringer Ingelheim.

## Publisher’s note

All claims expressed in this article are solely those of the authors and do not necessarily represent those of their affiliated organizations, or those of the publisher, the editors and the reviewers. Any product that may be evaluated in this article, or claim that may be made by its manufacturer, is not guaranteed or endorsed by the publisher.
